# Supplementary material for: Intention to have the seasonal influenza vaccination during the COVID-19 pandemic among eligible adults in the UK: a cross-sectional survey
Source: BMJ Open. 2021 Jul 13;11(7):e049369. doi: 10.1136/bmjopen-2021-049369 (PMC8282414; doi:10.1136/bmjopen-2021-049369)
Supplement: Supplementary data [file bmjopen-2021-049369supp001.pdf]

CoVAccS Survey 1 - Final

Start of Block: Information about the research

Q1.2 **Covid-19 Vaccination Acceptability Study – CoVAccS**
Survey exploring public attitudes towards a possible coronavirus (Covid-19) vaccination **Participant Information** We are interested in understanding how people feel about a possible coronavirus (Covid-19) vaccination and whether you would be likely to have the vaccine if one becomes available to you. We would be very grateful if you would complete this 3-part survey. The first part contains some questions about you, the second part contains questions about the coronavirus illness, the third part contains questions about a possible coronavirus vaccination, whether you would have such a vaccination and some general questions about your thoughts regarding vaccination and healthcare. To assist us with this, we would be grateful if you would complete this survey. It should take no more than 20 minutes. Please note that all responses will be anonymous. What will I need to do? If you decide to take part, you should tick the consent box at the bottom of this page and then complete the questionnaire. Please ensure that you have answered all questions on a page before moving on to the next one. Please note that once you have completed the final page you will be unable to withdraw your data as it is anonymous. Will my taking part in the study be kept confidential? Yes. The survey is anonymous (we don't ask for your name or any identifying information). What will happen to the results of the research study? We intend to publish the results of this study in scientific journals. We will also present it at scientific conferences and we may also pass the results of the research to relevant policy makers. This will contribute to the debate around vaccination policy. You will not be identified in any research presentation or publication as the study is completed anonymously. The data you have provided will be stored on a password protected computer and laptop. On completion of the project the data will be stored indefinitely in an online repository such as the Open Science Framework to which access will be open. You will not be identifiable from the data since your participation is entirely anonymous. Who is conducting the research? The research is being conducted by a team from Keele University, King's College London, and Public Health England. Who has reviewed the study? All research conducted by Keele University is looked at by an independent group of people, called a Research Ethics Committee, to protect your interests. This study has been reviewed and approved by Keele's Research Ethics Committee. What if there is a problem? If you have a concern about any aspect of this study, you may wish to speak to the principal investigator, Dr Sue Sherman, at Keele University at s.m.sherman@keele.ac.uk. Alternatively, you may wish to contact Dr Joseph Brooks (j.l.brooks@keele.ac.uk) who is the Director of Research in the School of Psychology at Keele University.

Q1.3
Consent agreement If you are happy to take part in this survey, please read the following statements: I confirm that I have read and understand the information above for this study I understand that my data will be anonymous and that my participation is voluntary I understand that I have the right to withdraw from the study at any time by closing the survey I agree to allow the data collected to be used for research projects related to this project Now please click on one of the options below:

○ I agree with the statements above and I am happy to take part in this study (1)

○ I do not wish to take part in this study (2)

End of Block: Information about the research

Start of Block: Does not consent

Q2.1
As you do not wish to participate in this study, please return your submission on Prolific by selecting the 'Stop without completing' button.

End of Block: Does not consent

Start of Block: Prolific ID

Q3.2
Before you start, please:

maximize your browser window;
switch off phone/e-mail/music and anything else distracting
and please enter your Prolific ID in the box below [it can be found at the top of this webpage or when going to your account info]:

____

End of Block: Prolific ID

Start of Block: Age check

Q4.2 How old are you?

- 17 or younger (1)
- 18 (2)
- Then options through to
- 100 (84)
- Older than 100 (104)

End of Block: Age check

Start of Block: Not eligible

Q5.1 Unfortunately you are not eligible to take part in this survey as you are under 18, please return your submission on Prolific by selecting the 'Stop without completing' button.

End of Block: Not eligible

Start of Block: Demographics

Q6.2 **PART 1: INFORMATION ABOUT YOU**

Q6.3 How would you describe your gender?

- Female (0)
- Male (1)
- Non-binary (2)
- Prefer to self-describe (please specify) (3) ________________
- Prefer not to say (-7)

Q6.4 Please choose one of the options below which best describes your ethnic group or background. These categories reflect the categories used in the Census 2011.

- ○ English/ Welsh/ Scottish/ Northern Irish/ British (1)
- ○ Irish (2)
- ○ Gypsy or Irish traveller (3)
- ○ Any other white background, please specify (4) ________________
- ○ White & Black Caribbean (5)
- ○ White and Black African (6)
- ○ White and Asian (7)
- ○ Any Other Mixed background, please specify (8) ________________
- ○ Indian (9)
- ○ Pakistani (10)
- ○ Bangladeshi (11)
- ○ Chinese (12)
- ○ Any other Asian background, please specify (13) ________________
- ○ African (14)
- ○ Caribbean (15)
- ○ Any other Black background, please specify (16) ________________
- ○ Arab (17)

○ Any other ethnic group, please specify (18)

____

○ Prefer not to say (-7)

Page Break

Q6.6 What is your religion?

○ No religion (1)

○ Christian (2)

○ Buddhist (3)

○ Hindu (4)

○ Jewish (5)

○ Muslim (6)

○ Sikh (7)

○ Any other religion, please describe (8)

____

○ Prefer not to say (-7)

Q6.7 What is the highest level of educational or professional qualification you have received?

- No formal qualifications (1)
- Youth training certificate/skillseekers (2)
- Recognised trade apprenticeship (3)
- Clerical and commercial (4)
- City & Guilds certificate (5)
- City & Guilds certificate – advanced (6)
- ONC (7)
- CSE grades 2–5 (8)
- CSE grade 1, GCE O level, GCSE, School Certificate (9)
- Scottish Ordinary/ Lower Certificate (10)
- GCE A level or Higher Certificate (11)
- Scottish Higher Certificate (12)
- Nursing or midwifery qualification (e.g. SEN, SRN, SCM, RGN) (13)
- Teaching qualification (not degree) (14)
- University diploma (15)
- University or CNAA first degree (e.g. BA, BSc, BEd) (16)
- University or CNAA higher degree (e.g. MSc, PhD) (17)
- Other technical, professional or higher qualification (18)
- Don't know (19)

○ Prefer not to say (-7)

Q6.8 Which ONE of the following best describes your current working situation?

○ Working full time (30 hours per week or more) (1)

○ Usually working full time (30 hours per week or more), but currently furloughed (2)

○ Working part time (8–29 hours per week) (3)

○ Usually working part time (8–29 hours per week), but currently furloughed (4)

○ Stay-at-home parent (5)

○ Unemployed (6)

○ Retired (7)

○ Student (8)

○ Other (9)

○ Don't know (10)

○ Prefer not to say (-7)

Q6.9 In which of the following categories would you place your total household income from all sources before tax and any other deductions?

- ○ Under £10,000 (1)
- ○ £10,000–£19,999 (2)
- ○ £20,000–£29,999 (3)
- ○ £30,000–£39,999 (4)
- ○ £40,000–£49,999 (5)
- ○ £50,000–£74,999 (6)
- ○ £75,000 or over (7)
- ○ Don't know (8)
- ○ Prefer not to say (-7)

Page Break

Sherman SM, *et al. BMJ Open* 2021; 11:e049369. doi: 10.1136/bmjopen-2021-049369

Q6.11 Where in the UK do you live?

- East Midlands (1)
- East of England (2)
- London (3)
- North East (4)
- North West (5)
- Northern Ireland (6)
- Scotland (7)
- South East (8)
- South West (9)
- Wales (10)
- West Midlands (11)
- Yorkshire and the Humber (12)
- Prefer not to say (-7)

Q6.12 How many people live in your household including yourself?

- 1 (1)
- 2 (2)
- 3–4 (3)
- 5–6 (4)
- 7 or more (5)
- Prefer not to say (-7)

Q6.13 The colour test is simple, when asked for your favourite colour you must enter the word purple in the text box below.

Based on the text you read above, what colour have you been asked to enter?

____

Q6.14 Do any of the following apply to you or someone else in your household?

	You			Someone else in your household		
	Yes (1)	No (0)	Prefer not to say (-7)	Yes (1)	No (0)	Prefer not to say (-7)

Received a letter from the NHS recommending that extra precautions against coronavirus are taken (this is called 'shielding') (Q6.11_1)	○	○	○	○	○	○
A lung condition (such as asthma, COPD, emphysema or bronchitis) (Q6.11_2)	○	○	○	○	○	○
Heart disease (such as heart failure) (Q6.11_3)	○	○	○	○	○	○
Chronic kidney disease (Q6.11_4)	○	○	○	○	○	○
Liver disease (such as hepatitis) (Q6.11_5)	○	○	○	○	○	○
A condition affecting the brain or nerves (such as Parkinson's disease, motor neurone disease, multiple sclerosis or cerebral palsy) (Q6.11_6)	○	○	○	○	○	○
Diabetes (Q6.11_7)	○	○	○	○	○	○

Sherman SM, *et al. BMJ Open* 2021; 11:e049369. doi: 10.1136/bmjopen-2021-049369

A condition that means there is a high risk of getting infections (Q6.11_8)	○	○	○	○	○	○
Taking medicine that can affect the immune system (such as steroids) (Q6.11_9)	○	○	○	○	○	○
Classified as very obese (a body mass index (BMI) of 40 or above) (Q6.11_10)	○	○	○	○	○	○
Pregnant (Q6.11_11)	○	○	○	○	○	○

Q6.15 Do any of the following apply to you?

	You		
	Yes (1)	No (0)	Prefer not to say (-7)

Received a letter from the NHS recommending that extra precautions against coronavirus are taken (this is called 'shielding') (Q6.12_1)	○	○	○
A lung condition (such as asthma, COPD, emphysema or bronchitis) (Q6.12_2)	○	○	○
Heart disease (such as heart failure) (Q6.12_3)	○	○	○
Chronic kidney disease (Q6.12_4)	○	○	○
Liver disease (such as hepatitis) (Q6.12_5)	○	○	○
A condition affecting the brain or nerves (such as Parkinson's disease, motor neurone disease, multiple sclerosis or cerebral palsy) (Q6.12_6)	○	○	○
Diabetes (Q6.12_7)	○	○	○
A condition that means there is a high risk of getting infections (Q6.12_8)	○	○	○
Taking medicine that can affect the immune system (such as steroids) (Q6.12_9)	○	○	○
Classified as very obese (a body mass index (BMI) of 40 or above) (Q6.12_10)	○	○	○

Sherman SM, *et al. BMJ Open* 2021; 11:e049369. doi: 10.1136/bmjopen-2021-049369

Pregnant (Q6.12_11)	○	○	○

Page Break

Q6.17 Please could you indicate if you work in any of the following sectors or roles? Please include any voluntary work. Please tick all that apply.

- ▢ Health or social care (e.g. doctors, nurses, midwives, paramedics, social workers, care workers; or work as part of the health and social care supply chain, including producers and distributers of medicines and medical equipment) (1)
- ▢ Education and childcare (e.g. teaching and support staff, childminders, social workers, specialist education professionals) (2)
- ▢ Key public services (e.g. the justice system, religious staff, charities delivering frontline services, journalists, broadcasters, undertakers) (3)
- ▢ Local and national government in a role essential to continuous provision of essential services (e.g the payment of benefits, or processing of new benefit applications) (4)
- ▢ Food and essential goods (e.g. food production, processing, distribution, sale and delivery, as well as those essential to the provision of other key goods such as hygienic or veterinary medicine) (5)
- ▢ Public safety and national security (e.g. police and support staff, Ministry of Defence civilians, contractor and armed forces, fire and rescue service employees, National Crime Agency staff, border security staff, prison and probation staff and other national security roles) (6)
- ▢ Transport (e.g. air, water, road and rail passenger and freight transport modes) (7)
- ▢ Utilities, communication and financial services (e.g. banks, building societies and financial market infrastructure; the oil, gas, electricity and water sectors; information technology and data infrastructure sector; civil nuclear, chemicals, telecommunications, network operations, field engineering, call centre staff, IT and data infrastructure, 999 and

111 critical services, postal services and delivery, payments providers and waste disposal) (8)

☐ None of the above (9)

☐ Prefer not to say (-7)

Q6.18 Last winter, did you have a vaccination for seasonal flu?

○ Yes (1)

○ No (0)

○ Don't know (2)

○ Prefer not to say (-7)

End of Block: Demographics

Start of Block: Pre-survey statement

Q7.2 Over the next few pages we will ask you a series of questions about the coronavirus illness and a possible vaccination. We are interested to know your personal opinion about these topics.

Please click on 'NEXT' when you are ready to continue.

End of Block: Pre-survey statement

Start of Block: Questions about Covid-19 the illness 1

Q8.2 **PART 2: YOUR THOUGHTS ABOUT THE CORONAVIRUS (COVID-19) ILLNESS**

Q8.3
To what extent do you think coronavirus poses a risk to people in the UK?

- ○ Major risk (5)
- ○ Significant risk (4)
- ○ Moderate risk (3)
- ○ Minor risk (2)
- ○ No risk at all (1)
- ○ Don't know (0)

Q8.4
To what extent do you think coronavirus poses a risk to you personally?

- ○ Major risk (5)
- ○ Significant risk (4)
- ○ Moderate risk (3)
- ○ Minor risk (2)
- ○ No risk at all (1)
- ○ Don't know (0)

Q8.5 Do you believe you have had, or currently have, coronavirus? (Please select the one option that BEST applies to you)

- ○ I have definitely had it or definitely have it now (4)
- ○ I have probably had it or probably have it now (3)
- ○ I have probably not had it and probably don’t have it now (2)
- ○ I have definitely not had it and definitely don’t have it now (1)
- ○ Don’t know (0)
- ○ Prefer not to say (-7)

Q8.6 Do you personally know anyone (excluding yourself) who has had coronavirus?

- ○ Yes (1)
- ○ No (0)
- ○ Don't know (0)
- ○ Prefer not to say (-7)

End of Block: Questions about Covid-19 the illness 1

Start of Block: Questions about Covid-19 the illness 2

Q9.2 PART 2: YOUR THOUGHTS ABOUT THE CORONAVIRUS (COVID-19) ILLNESS

Q9.3 Please indicate the extent to which you agree or disagree with the following statements by ticking a number between 0 and 10, where **0 means ‘strongly disagree’ and 10 means ‘strongly agree':**

Sherman SM, *et al. BMJ Open* 2021; 11:e049369. doi: 10.1136/bmjopen-2021-049369

Q9.4 I am worried about catching coronavirus
Q9.5 I believe that coronavirus would be a mild illness for me
Q9.6 Too much fuss is being made about the risk of coronavirus
Q9.7 We are all responsible for reducing the spread of the coronavirus
Q9.8 It's important that you pay attention to this study, so please select 0 ('strongly disagree') for this item
Q9.9 I believe I am immune to coronavirus
Q9.10 The coronavirus pandemic has had a big impact on my life
Q9.11 I trust the NHS to manage the coronavirus pandemic in the UK
Q9.12 I trust the Government to manage the coronavirus pandemic in the UK

End of Block: Questions about Covid-19 the illness 2

Start of Block: Questions about the Covid-19 vaccination 1

Q10.2 **PART 3: YOUR THOUGHTS ABOUT A CORONAVIRUS (COVID-19) VACCINATION**

For the following questions, please imagine that a coronavirus vaccine is widely available.

Q10.3 When a coronavirus vaccination becomes available to you, how likely is it that you will have one? Please select a number between 0 and 10, where **0 means 'extremely unlikely' and 10 means 'extremely likely'**:

End of Block: Questions about the Covid-19 vaccination 1

Start of Block: Questions about the Covid-19 vaccination 2

Q11.2 **PART 3: YOUR THOUGHTS ABOUT A CORONAVIRUS (COVID-19) VACCINATION**

For the following questions, please imagine that a coronavirus vaccine is widely available.

Q11.3 Please indicate the extent to which you agree or disagree with the following statements by ticking a number between 0 and 10, where **0 means 'strongly disagree' and 10 means 'strongly agree':**

Sherman SM, *et al. BMJ Open* 2021; 11:e049369. doi: 10.1136/bmjopen-2021-049369

Q11.4 A coronavirus vaccination should be mandatory for everyone who is able to have it
Q11.5 Without a coronavirus vaccine, I am likely to catch coronavirus
Q11.6 If I get a coronavirus vaccination, I will be protected against coronavirus
Q11.7 If I don't get a coronavirus vaccination and end up getting coronavirus, I would regret not getting the vaccination
Q11.8 It would be very easy for me to have a coronavirus vaccination
Q11.9 A coronavirus vaccination could give me coronavirus
Q11.10 It's important that you pay attention to this study, so please select 10 ('strongly agree') for this item
Q11.11 I would be worried about experiencing side effects from a coronavirus vaccination

End of Block: Questions about the Covid-19 vaccination 2

Start of Block: Questions about the Covid-19 vaccination 3

Q12.2 PART 3: YOUR THOUGHTS ABOUT A CORONAVIRUS (COVID-19) VACCINATION

For the following questions, please imagine that a coronavirus vaccine is widely available.

Q12.3 Please indicate the extent to which you agree or disagree with the following statements by ticking a number between 0 and 10, where **0 means 'strongly disagree' and 10 means 'strongly agree':**

Q12.4 I might regret getting a coronavirus vaccination if I later experienced side effects from the vaccination
Q12.5 A coronavirus vaccination will be too new for me to be confident about getting vaccinated
Q12.6 Most people will get a coronavirus vaccination
Q12.7 Other people like me will get a coronavirus vaccination
Q12.8 In general, vaccination is a good thing
Q12.9 I am afraid of needles
Q12.10 If I were vaccinated, I think I would not need to follow social distancing and other restrictions for coronavirus
Q12.11 I know enough about the coronavirus illness to make an informed decision about whether or not to get vaccinated
Q12.12 I know enough about the coronavirus vaccine to make an informed decision about whether or not to get vaccinated

End of Block: Questions about the Covid-19 vaccination 3

Start of Block: Questions about the Covid-19 vaccination 4

Q13.2 PART 3: YOUR THOUGHTS ABOUT A CORONAVIRUS (COVID-19) VACCINATION

For the following questions, please imagine that a coronavirus vaccine is widely available.

Q13.3 Please indicate the extent to which you agree or disagree with the following statements by ticking a number between 0 and 10, where **0 means ‘strongly disagree’ and 10 means ‘strongly agree':**

Q13.4 Only people who are at risk of serious illness from coronavirus need to be vaccinated
Q13.5 My family would approve of my having a coronavirus vaccination
Q13.6 My friends would approve of my having a coronavirus vaccination
Q13.7 If a coronavirus vaccination were recommended by the Government, I would get vaccinated
Q13.8 If a coronavirus vaccination were recommended by a health care professional (e.g. GP or nurse), I would get vaccinated
Q13.9 Widespread coronavirus vaccination is just a way to make money for vaccine manufacturers
Q13.10 A coronavirus vaccine will allow us to get back to ‘normal’
Q13.11 There would be no point in having the coronavirus vaccination unless I could go back to my normal life

End of Block: Questions about the Covid-19 vaccination 4

Start of Block: Questions about the Covid-19 vaccination 5

Q14.2 PART 3: YOUR THOUGHTS ABOUT A CORONAVIRUS (COVID-19) VACCINATION

For the following questions, please imagine that a coronavirus vaccine is widely available.

Q14.3 As far as you know, would your employer want you to have the coronavirus vaccination?

- ○ Yes (0)
- ○ No (1)
- ○ Don't know (2)
- ○ Not applicable (11)

Q14.4 As far as you know, is there currently a widely-available vaccination to protect against coronavirus?

- ○ Yes (1)
- ○ No (0)
- ○ Don't know (2)
- ○ Prefer not to say (-7)

Q14.5
This winter, how likely is it that you will have the seasonal flu vaccination? Please select a number between 0 and 10, where **0 means 'extremely unlikely' and 10 means 'extremely likely'**:

End of Block: Questions about the Covid-19 vaccination 5

Start of Block: Thank you

Q15.2 Thank you for taking part in this survey.

If you have any questions about the survey, please contact Dr Sue Sherman (s.m.sherman@keele.ac.uk).

If you have questions about coronavirus (Covid-19), please visit the NHS website: https://www.nhs.uk/conditions/coronavirus-covid-19/ or the government website: https://www.gov.uk/coronavirus.

End of Block: Thank you
