# Supplementary material for: Intention to have the seasonal influenza vaccination during the COVID-19 pandemic among eligible adults in the UK: a cross-sectional survey
Source: BMJ Open. 2021 Jul 13;11(7):e049369. doi: 10.1136/bmjopen-2021-049369 (PMC8282414; doi:10.1136/bmjopen-2021-049369)
Supplement: Author's manuscript [file bmjopen-2021-049369.draft_revisions.pdf]

BMJ Open is committed to open peer review. As part of this commitment we make the peer review history of every article we publish publicly available.

When an article is published we post the peer reviewers' comments and the authors' responses online. We also post the versions of the paper that were used during peer review. These are the versions that the peer review comments apply to.

The versions of the paper that follow are the versions that were submitted during the peer review process. They are not the versions of record or the final published versions. They should not be cited or distributed as the published version of this manuscript.

BMJ Open is an open access journal and the full, final, typeset and author-corrected version of record of the manuscript is available on our site with no access controls, subscription charges or pay-per-view fees (http://bmjopen.bmj.com).

If you have any questions on BMJ Open's open peer review process please email info.bmjopen@bmj.com

BMJ Open

Intention to have the seasonal influenza vaccination during the COVID-19 pandemic among eligible adults in the UK: A cross-sectional survey

Journal:	*BMJ Open*
Manuscript ID	bmjopen-2021-049369
Article Type:	Original research
Date Submitted by the Author:	22-Jan-2021
Complete List of Authors:	Sherman, Susan; Keele University<br>Sim, Julius; Keele University<br>Cutts, Megan; Keele University<br>Dasch, Hannah; King's College London<br>Smith, Louise; King's College London<br>Amlot, Richard; Public Health England Porton<br>Rubin, GJ; King's College London<br>Sevdalis, Nick; King's College London
Keywords:	COVID-19, Public health < INFECTIOUS DISEASES, PREVENTIVE MEDICINE, PUBLIC HEALTH

SCHOLARONE™
Manuscripts

I, the Submitting Author has the right to grant and does grant on behalf of all authors of the Work (as defined in the below author licence), an exclusive licence and/or a non-exclusive licence for contributions from authors who are: i) UK Crown employees; ii) where BMJ has agreed a CC-BY licence shall apply, and/or iii) in accordance with the terms applicable for US Federal Government officers or employees acting as part of their official duties; on a worldwide, perpetual, irrevocable, royalty-free basis to BMJ Publishing Group Ltd ("BMJ") its licensees and where the relevant Journal is co-owned by BMJ to the co-owners of the Journal, to publish the Work in this journal and any other BMJ products and to exploit all rights, as set out in our licence.

The Submitting Author accepts and understands that any supply made under these terms is made by BMJ to the Submitting Author unless you are acting as an employee on behalf of your employer or a postgraduate student of an affiliated institution which is paying any applicable article publishing charge ("APC") for Open Access articles. Where the Submitting Author wishes to make the Work available on an Open Access basis (and intends to pay the relevant APC), the terms of reuse of such Open Access shall be governed by a Creative Commons licence – details of these licences and which Creative Commons licence will apply to this Work are set out in our licence referred to above.

Other than as permitted in any relevant BMJ Author's Self Archiving Policies, I confirm this Work has not been accepted for publication elsewhere, is not being considered for publication elsewhere and does not duplicate material already published. I confirm all authors consent to publication of this Work and authorise the granting of this licence.

For peer review only

Title: Intention to have the seasonal influenza vaccination during the COVID-19 pandemic among eligible adults in the UK: A cross-sectional survey

Authors: Susan M. Sherman (https://orcid.org/0000-0001-6708-3398)[1], Julius Sim (https://orcid.org/0000-0002-1816-1676)[2], Richard Amlôt[3,4], Megan Cutts[1], Hannah Dasch[5,6], G James Rubin (https://orcid.org/0000-0002-4440-0570)[3,5], Nick Sevdalis (https://orcid.org/0000-0001-7560-8924)[5,6], Louise E. Smith (https://orcid.org/0000-0002-1277-2564)[3,5]

Affiliations

1. Keele University, School of Psychology, Keele, UK

2. Keele University, School of Medicine, Keele, UK

3. NIHR Health Protection Research Unit in Emergency Preparedness and Response

4. Public Health England, Behavioural Science Team, Emergency Response Department Science and Technology

5. King's College London, Institute of Psychiatry, Psychology and Neuroscience, London, UK

6. King's College London, Centre for Implementation Science

Corresponding author: Susan Mary Sherman, School of Psychology, Keele University, Keele, Staffs, ST5 5BG, UK, s.m.sherman@keele.ac.uk, 01782 733384

For peer review only - http://bmjopen.bmj.com/site/about/guidelines.xhtml

Abstract

Objective: To investigate the likelihood of having the seasonal influenza vaccination during the COVID-19 pandemic in individuals who were eligible to receive it.

Design: We conducted a cross-sectional online survey in July 2020. We included predictors informed by previous research: sociodemographic variables; uptake of influenza vaccine last winter; and beliefs about vaccination.

Participants: 570 participants (mean age: 53.07, 56.3% female, 87% white) who were eligible for the free seasonal influenza vaccination in the UK.

Results: We used logistic regression to investigate variables associated with intention to receive a seasonal influenza vaccine in 2020/21. 59.7% of our sample indicated they were likely to have the seasonal influenza vaccination, 22.1% reported being unlikely to have the vaccination and 18.2% were unsure. A positive attitude to vaccination in general predicted intention to have the influenza vaccine this year (95% CI 1.19 to 1.77, p<.001) but the strongest predictor of intention was previous influenza vaccination behaviour (95% CI 78.04 to 994.46, p<.001).

Conclusions: Previous research suggests that increasing uptake of the influenza vaccination may help contain a COVID-19 outbreak, so steps need to be taken to convert intention into behaviour and to reach those individuals who reported being unlikely or unsure about having the vaccine.

Keywords: influenza; vaccine; intention; COVID-19; coronavirus

Article Summary:

Strengths and limitations of this study

- First study to explore seasonal influenza vaccine acceptability in the UK during the COVID-19 pandemic
- Comprehensive demographic information was collected to facilitate statistical analysis of eligible subgroups
- Survey measured intention rather than behaviour

Word count: 2039 (excluding abstract and references)

For peer review only

Introduction

As we are in the midst of the seasonal influenza season in the global North, we need to understand influences on intention to have the influenza vaccination this year in order to maximize uptake and help contain subsequent COVID-19 and other infectious disease outbreaks. We report findings from a survey conducted in July 2020 in the UK, which explored participants' likelihood of having the seasonal influenza vaccination this winter (2020–2021).

The COVID-19 pandemic was declared on March 11th, 2020. While the first wave of the pandemic missed most of the influenza season in the Northern hemisphere, a second wave is likely to overlap with the 2020–2021 season [1]. Healthcare systems come under considerable strain during a typical influenza season; this could be compounded by a large number of COVID-19 cases this winter. Recent research has modelled the impact of mass influenza vaccination on the spread of COVID-19 should such an overlap occur, and the findings suggest that increasing uptake of the influenza vaccination would facilitate efforts to contain a COVID-19 outbreak [2]. However, increasing, or even maintaining, levels of influenza vaccination may be problematic if reduced uptake patterns seen already in other vaccines also hold for the influenza vaccine. For example, the uptake of the measles-mumps-rubella (MMR) vaccine in England became 19.8% lower in the 3 weeks after full physical distancing measures were introduced in March than it was in 2019 [3].

The influenza season in the UK runs from December until March each year and the national vaccination program starts from September. At the time of data collection, eligibility for the free vaccine through the National Health Service (NHS) was the same as in previous years, being available to children aged two to eleven, adults over 65, pregnant women, health and social care workers, individuals aged 6 months to 65 years who are in clinical at-risk groups (many of which coincide with the COVID-19 at-risk groups), those living in a residential or nursing home, and anyone who is the main carer of an older or disabled person. For the 2020–2021 influenza season, the free vaccination program has been extended to all adults aged 50 or older as well as to anyone living with someone who is at high risk from coronavirus. The vaccination is also available privately for a charge through primary care and pharmacies to the rest of the population. Despite the wide availability of a free vaccine for eligible individuals, uptake varies across the different categories of eligibility; for example, in the 2019–2020 season 72.4% of 65+ adults in England were vaccinated compared to 44.9% of individuals aged 6 months to 65 in clinical at-risk groups [4].

In order to protect people ahead of and during the influenza season, it is helpful to understand intention to have a seasonal influenza vaccination during the COVID-19 pandemic. To this end, we explored participants' likelihood of having the seasonal influenza vaccination as part of a larger

For peer review only - http://bmjopen.bmj.com/site/about/guidelines.xhtml

cross-sectional study investigating attitudes towards a potential COVID-19 vaccination [5]. Previous research exploring factors associated with seasonal influenza vaccination uptake has identified a range of factors that might influence seasonal influenza uptake [6,7]. In this study we focused on socio-demographic factors such as age, ethnicity, and gender, general attitude towards vaccination, fear of needles, and past behavior (whether individuals had previously had a seasonal influenza vaccination).

Materials and Methods

Ethical approval for this study was granted by Keele University's Research Ethics Committee (reference: PS-200129). A nationally representative quota sample of 1500 UK adults (quotas set on age, gender and ethnicity) were recruited through Prolific's online research panel to complete a cross-sectional survey between 14th and 17th July 2020. Participants were included in this study if they were eligible to receive the free influenza vaccine through the NHS at the time of data collection (aged 65 years or over, pregnant, working in health or social care, or in a clinical risk group)[1]. After providing informed consent, participants were asked to complete the survey which included sociodemographic questions (e.g., age, gender, ethnicity, employment status, highest educational or professional qualification), clinical questions (e.g., whether they or someone else in their household (if applicable) had a chronic illness that made them clinically vulnerable to serious illness from COVID-19), questions about COVID-19 (e.g., whether they were worried about catching coronavirus), and questions about a possible COVID-19 vaccination (e.g., whether they thought most people would get a coronavirus vaccination). We also asked participants to what extent they agreed that 'in general, vaccination is a good thing' and to what degree they were 'afraid of needles' (both on an eleven-point scale from "strongly disagree" to "strongly agree"), and if they had been vaccinated for seasonal influenza last winter (yes/no). The outcome measure for this study, vaccination intention, was measured by asking participants how likely they would be to have the seasonal influenza vaccine this winter (eleven-point scale, from "extremely unlikely" to "extremely likely"). Full details of the wider study, including survey methodology, are reported elsewhere [5].

Since eligibility for the free influenza vaccine in 2020-2021 was widened after data collection, as a sensitivity analysis, we re-ran analyses using these broader criteria.

Patient and public involvement

[1] We did not collect data on whether participants were living in a care home or whether they were a main carer and so these eligibility criteria are not explicitly represented in our analysis.

For peer review only - http://bmjopen.bmj.com/site/about/guidelines.xhtml

Patients or the public were not involved in the design, or conduct, or reporting or dissemination plans of this research.

Results

At the time of data collection, 570 individuals in our sample were eligible for the free influenza vaccine. The distribution of influenza vaccination intention was bimodal, with the majority of responses clustering at both ends of the scale. We therefore dichotomized this variable as 0–2 = 'no' (*n* = 126; 22.1%) and 8–10 = 'yes' (*n* = 340; 59.7%) on the 0–10 scale; the 104 (18.2%) indeterminate cases were not analyzed further. In the 466 respondents who expressed a clear intention either to have or not to have the seasonal influenza vaccine, we investigated variables associated with intention to receive the vaccine in 2020–2021 using logistic regression analysis, and included predictors informed by previous research [6,7]: sociodemographic variables; uptake of influenza vaccine last winter; and beliefs about vaccination (value of vaccination in general; not afraid of needles). These variables are shown in Table 1 and the results of the regression analysis are shown in Table 2.

Table 1. Characteristics and attitudes of those respondents who expressed a clear intention either to have or not to have the influenza vaccine (*n*=466). Values are *n* (%) except where stated otherwise.

**Variable**	
Age (years): mean (SD); range	53.07 (16.86); 18–87
Gender[a]	
Male	203 (43.7)
Female	262 (56.3)
Ethnicity[b]	
White	403 (87.0)
Non-white	60 (13.0)
Qualifications	
Degree equivalent or higher	231 (49.6)
Other	235 (50.4)
Working[c]	
Part-time	58 (12.5)
Full-time	136 (29.3)
Not working/other[d]	270 (58.2)

For peer review only - http://bmjopen.bmj.com/site/about/guidelines.xhtml

Key worker	
Yes	175 (37.6)
No	291 (62.4)
Influenza vaccination last winter[a]	
Yes	299 (64.3)
No	166 (35.7)
In general, vaccination is a good thing (0–10 scale): mean (SD)[e]	8.78 (2.09)
I am afraid of needles (0–10 scale): mean (SD)[e]	2.14 (3.17)

[a] 1 missing value; [b] 3 missing values; [c] 2 missing values; [d] includes 30 unemployed, 36 furloughed and 157 retired; [e] these variables showed a marked skew.

For peer review only

Table 2. Logistic regression analysis of variables associated with intention to receive a seasonal influenza vaccine. Reference categories for the odds ratios are shown where appropriate. Statistical significance was set at $p \leq .05$.

	**Odds ratio**	**95% confidence interval**	***p* value**
Age (years)	1.02	0.99, 1.04	.190
Gender (reference: female)	1.24	0.60, 2.59	.565
Ethnicity – white (reference: black and minority ethnic)	0.60	0.23, 1.53	.281
Qualifications – degree equivalent or higher (reference: other)	1.52	0.72, 3.22	.278
Working (reference: not working/other)			
Part-time	1.12	0.39, 3.21	.830
Full-time	1.51	0.64, 3.56	.342
Key worker (reference: not key worker)	0.59	0.27, 1.31	.197
Influenza vaccination last winter (reference: no vaccination)	278.58	78.04, 994.46	<.001
In general, vaccination is a good thing (0–10 scale)	1.45	1.19, 1.77	<.001
I am afraid of needles (0–10 scale)	0.98	0.89, 1.09	.757

n = 460 (6 cases with missing data on one or more variables were not analyzed). Nagelkerke R^2 = .760.

For peer review only - http://bmjopen.bmj.com/site/about/guidelines.xhtml

A positive attitude to vaccination and previous vaccination behavior were significant predictors of intention to have the influenza vaccine in 2020–2021. As indicated by the large odds ratio, previous influenza vaccination behavior was a markedly stronger predictor.

In our sample, 1003 respondents met the broadened eligibility criteria for the free influenza vaccine in 2020–2021. Of these, 491 (49.0%) respondents expressed a clear intention to have the vaccine, 291 (29.0%) a clear intention not to have the vaccine, and there were 221 (22.0%) indeterminate cases. As before, the indeterminate cases were not analyzed. The results of the sensitivity analysis are shown in Table 3.

Table 3. Logistic regression analysis of variables associated with intention to receive a seasonal influenza vaccine, using the broadened eligibility criteria introduced in 2020–2021. Reference categories for the odds ratios are shown where appropriate. Statistical significance was set at $p \leq .05$.

	**Odds ratio**	**95% confidence interval**	***p* value**
Age (years)	1.02	1.00, 1.04	.046
Gender (reference: female)	1.24	0.76, 2.01	.388
Ethnicity – white (reference: black and minority ethnic)	0.75	0.36, 1.56	.443
Qualifications – degree equivalent or higher (reference: other)	1.00	0.62, 1.59	.983
Working (reference: not working/other)			
Part-time	0.80	0.40, 1.58	.519
Full-time	1.03	0.58, 1.81	.929
Key worker (reference: not key worker)	0.93	0.55, 1.57	.776
Influenza vaccination last winter (reference: no vaccination)	281.78	95.35, 832.72	<.001
In general, vaccination is a good thing (0–10 scale)	1.54	1.31, 1.81	<.001
I am afraid of needles (0–10 scale)	0.98	0.91, 1.05	.529

n = 774 (8 cases with missing data on one or more variables were not analyzed). Nagelkerke R^2 = .716.

Compared to the main analysis, a somewhat smaller percentage of respondents indicated a clear intention to be vaccinated (49.0% versus 59.7%), and correspondingly a larger percentage not to be vaccinated (29.0% versus 22.1%). However, the odds ratios were of a similar magnitude to those in the main analysis and with similar associated *p* values (though with the larger sample size, the odds ratio for age became significant), suggesting that whilst the broader eligibility criteria may influence

For peer review only - http://bmjopen.bmj.com/site/about/guidelines.xhtml

the percentage of individuals intending to be vaccinated against influenza, they have little effect on the predictors of such vaccination behavior.

Discussion

These findings strongly suggest that individuals who had the influenza vaccine last year are likely to intend to have it again this year, and this is consistent with findings from the H1N1 influenza pandemic [6]. However, there are still key issues to address. Vaccination intention across all those individuals in our sample who were eligible for the vaccine (59.7%) at the time of data collection was slightly lower than the reported uptake from last year (64.3%). Furthermore, it is likely that actual uptake will be lower than intention as a result of the intention-behavior gap [8], making it important that efforts are made to convert positive intentions into uptake. This might be achieved through appropriate messaging and special arrangements for vaccine delivery, particularly for those who might be shielding or at higher risk from COVID-19 and reluctant to attend their GP surgery. Both approaches are also likely to be needed to motivate those individuals who have not previously had the influenza vaccine and those individuals who are eligible for free vaccination but who were among respondents in our sample who indicated they definitely did not intend to be vaccinated (22.1%) or were unsure (18.2%).

The NHS is often overwhelmed during the influenza season, needing, for example, to cancel routine operations. The extension of the influenza vaccination program to people aged 50 years and over and to those living with someone who is at high risk from coronavirus may also help decrease the burden of influenza in winter 2020–2021. During the first wave of the COVID-19 pandemic in the UK, which occurred outside the influenza season, there was sufficient concern about the NHS's ability to cope that 10 new 'Nightingale' hospitals were built. Increasing uptake of the seasonal influenza vaccine in a timely fashion will relieve pressure on the service. If this is to be successful, strategies to achieve this increase need to be implemented now.

For peer review only - http://bmjopen.bmj.com/site/about/guidelines.xhtml

Data statement: Survey items and dataset are available from: DOI 10.17605/OSF.IO/94856.

Conflict of interest: NS is the director of the London Safety and Training Solutions Ltd, which offers training in patient safety, implementation solutions and human factors to healthcare organisations. The other authors have no conflicts of interest to declare.

Funding statement: Data collection was funded by a Keele University Faculty of Natural Sciences Research Development award to SS, JS and NS, and a King's Together Rapid COVID-19 award granted jointly to LS, GJR, RA, NS, SS and JS. LS, RA and GJR are supported by the National Institute for Health Research Health Protection Research Unit (NIHR HPRU) in Emergency Preparedness and Response, a partnership between Public Health England, King's College London and the University of East Anglia. NS' research is supported by the National Institute for Health Research (NIHR) Applied Research Collaboration (ARC) South London at King's College Hospital NHS Foundation Trust. NS is a member of King's Improvement Science, which offers co-funding to the NIHR ARC South London and comprises a specialist team of improvement scientists and senior researchers based at King's College London. Its work is funded by King's Health Partners (Guy's and St Thomas' NHS Foundation Trust, King's College Hospital NHS Foundation Trust, King's College London and South London and Maudsley NHS Foundation Trust), Guy's and St Thomas' Charity and the Maudsley Charity. The views expressed are those of the authors and not necessarily those of the NIHR, the charities, Public Health England or the Department of Health and Social Care.

Author Contributions

Conceptualization, LS; Methodology, RA, MC, HD, GJR, NS, SS, JS, LS; Software, SS; Formal Analysis, JS; Writing – Original Draft Preparation, SS; Writing – Review & Editing, RA, MC, HD, GJR, NS, SS, JS, LS; Funding Acquisition, RA, GJR, NS, SS, JS, LS.

For peer review only - http://bmjopen.bmj.com/site/about/guidelines.xhtml

References

1. Maltezou, H. C., Theodoridou, K., & Poland, G. (2020). Influenza immunization and COVID-19. *Vaccine,* 38(39), 6078–6079.

2. Li, Q., Tang, B., Bragazzi, N. L., Xiao, Y., & Wu, J. (2020). Modeling the impact of mass influenza vaccination and public health interventions on COVID-19 epidemics with limited detection capability. *Mathematical Biosciences*, 325, 108378.

3. McDonald, H. I., Tessier, E., White, J. M., Woodruff, M., Knowles, C., Bates, C., ... & Yarwood, J. (2020). Early impact of the coronavirus disease (COVID-19) pandemic and physical distancing measures on routine childhood vaccinations in England, January to April 2020. *Eurosurveillance,* 25(19), 2000848.

4. Public Health England (2020). *Surveillance of influenza and other respiratory viruses in the UK: Winter 2019 to 2020.* London.

5. Sherman, S. M., Smith, L. E., Sim, J., Amlôt, R., Cutts, M., Dasch, H., Rubin, G. R., & Sevdalis, N. (2020). COVID-19 vaccination intention in the UK: Results from the COVID-19 Vaccination Acceptability Study (CoVAccS), a nationally representative cross-sectional survey. *Human Vaccines & Immunotherapeutics.* DOI: 10.1080/21645515.2020.1846397

6. Bish, A., Yardley, L., Nicoll, A., & Michie, S. (2011). Factors associated with uptake of vaccination against pandemic influenza: a systematic review. *Vaccine,* 29(38), 6472-6484.

7. Wheelock A, Miraldo M, Thomson A, Vincent C, Sevdalis N. (2017). Evaluating the importance of policy amenable factors in explaining influenza vaccination: a cross-sectional multinational study. *BMJ Open*;7:e014668

8. Sniehotta, F. F., Scholz, U., & Schwarzer, R. (2005). Bridging the intention–behaviour gap: Planning, self-efficacy, and action control in the adoption and maintenance of physical exercise. *Psychology & Health*, 20(2), 143-160.

For peer review only

BMJ Open

Intention to have the seasonal influenza vaccination during the COVID-19 pandemic among eligible adults in the UK: A cross-sectional survey

Journal:	*BMJ Open*
Manuscript ID	bmjopen-2021-049369.R1
Article Type:	Original research
Date Submitted by the Author:	04-Mar-2021
Complete List of Authors:	Sherman, Susan; Keele University<br>Sim, Julius; Keele University<br>Amlot, Richard; Public Health England Porton<br>Cutts, Megan; Keele University<br>Dasch, Hannah; King's College London<br>Rubin, GJ; King's College London<br>Sevdalis, Nick; King's College London<br>Smith, Louise; King's College London
**Primary Subject Heading**:	Public health
Secondary Subject Heading:	Infectious diseases
Keywords:	COVID-19, Public health < INFECTIOUS DISEASES, PREVENTIVE MEDICINE, PUBLIC HEALTH

SCHOLARONE™
Manuscripts

I, the Submitting Author has the right to grant and does grant on behalf of all authors of the Work (as defined in the below author licence), an exclusive licence and/or a non-exclusive licence for contributions from authors who are: i) UK Crown employees; ii) where BMJ has agreed a CC-BY licence shall apply, and/or iii) in accordance with the terms applicable for US Federal Government officers or employees acting as part of their official duties; on a worldwide, perpetual, irrevocable, royalty-free basis to BMJ Publishing Group Ltd ("BMJ") its licensees and where the relevant Journal is co-owned by BMJ to the co-owners of the Journal, to publish the Work in this journal and any other BMJ products and to exploit all rights, as set out in our licence.

The Submitting Author accepts and understands that any supply made under these terms is made by BMJ to the Submitting Author unless you are acting as an employee on behalf of your employer or a postgraduate student of an affiliated institution which is paying any applicable article publishing charge ("APC") for Open Access articles. Where the Submitting Author wishes to make the Work available on an Open Access basis (and intends to pay the relevant APC), the terms of reuse of such Open Access shall be governed by a Creative Commons licence – details of these licences and which Creative Commons licence will apply to this Work are set out in our licence referred to above.

Other than as permitted in any relevant BMJ Author's Self Archiving Policies, I confirm this Work has not been accepted for publication elsewhere, is not being considered for publication elsewhere and does not duplicate material already published. I confirm all authors consent to publication of this Work and authorise the granting of this licence.

For peer review only

Title: Intention to have the seasonal influenza vaccination during the COVID-19 pandemic among eligible adults in the UK: A cross-sectional survey

Authors: Susan M. Sherman (https://orcid.org/0000-0001-6708-3398)[1], Julius Sim (https://orcid.org/0000-0002-1816-1676)[2], Richard Amlôt[3,4], Megan Cutts[1], Hannah Dasch[5,6], G James Rubin (https://orcid.org/0000-0002-4440-0570)[3,5], Nick Sevdalis (https://orcid.org/0000-0001-7560-8924)[5,6], Louise E. Smith (https://orcid.org/0000-0002-1277-2564)[3,5]

Affiliations

1. Keele University, School of Psychology, Keele, UK

2. Keele University, School of Medicine, Keele, UK

3. NIHR Health Protection Research Unit in Emergency Preparedness and Response

4. Public Health England, Behavioural Science Team, Emergency Response Department Science and Technology

5. King's College London, Institute of Psychiatry, Psychology and Neuroscience, London, UK

6. King's College London, Centre for Implementation Science

Corresponding author: Susan Mary Sherman, School of Psychology, Keele University, Keele, Staffs, ST5 5BG, UK, s.m.sherman@keele.ac.uk, 01782 733384

For peer review only

Abstract

Objective: To investigate the likelihood of having the seasonal influenza vaccination during the COVID-19 pandemic in individuals who were eligible to receive it.

Design: We conducted a cross-sectional online survey in July 2020. We included predictors informed by previous research, in the following categories: sociodemographic variables; uptake of influenza vaccine last winter; and beliefs about vaccination.

Participants: 570 participants (mean age: 53.07; 56.3% female, 87.0% white) who were eligible for the free seasonal influenza vaccination in the UK.

Results: 59.7% of our sample indicated they were likely to have the seasonal influenza vaccination, 22.1% reported being unlikely to have the vaccination and 18.2% were unsure. We used logistic regression to investigate variables associated with intention to receive a seasonal influenza vaccine in the 2020-2021 season. A positive attitude to vaccination in general predicted intention to have the influenza vaccine in 2020-2021 (odds ratio 1.45, 95% CI 1.19 to 1.77, p<.001) but the strongest predictor of intention was previous influenza vaccination behaviour (odds ratio 278.58, 95% CI 78.04 to 994.46, p<.001).

Conclusions: Previous research suggests that increasing uptake of the influenza vaccination may help contain a COVID-19 outbreak, so steps need to be taken to convert intention into behaviour and to reach those individuals who reported being unlikely or unsure about having the vaccine.

Keywords: influenza; vaccine; intention; COVID-19; coronavirus

Article Summary:

Strengths and limitations of this study

- First study to explore seasonal influenza vaccine acceptability in the UK during the COVID-19 pandemic
- Comprehensive demographic information was collected to facilitate statistical analysis of eligible subgroups
- Survey measured intention rather than behaviour

Word count: 2501 (excluding abstract and references)

For peer review only - http://bmjopen.bmj.com/site/about/guidelines.xhtml

Introduction

To maximize uptake and help contain subsequent COVID-19 and other infectious disease outbreaks, we need to understand influences on intention to have the influenza vaccination while COVID-19 is circulating. We report findings from a survey conducted in July 2020 in the UK, which explored participants' likelihood of having the seasonal influenza vaccination in 2020–2021.

The COVID-19 pandemic was declared on March 11th, 2020. While the first wave of the pandemic missed most of the influenza season in the Northern hemisphere, a second wave has overlapped with the 2020–2021 season [1]. Healthcare systems come under considerable strain during a typical influenza season; this has been compounded by a large number of COVID-19 cases. Recent research modelling the impact of mass influenza vaccination on the spread of COVID-19 should such an overlap occur suggest that increasing uptake of the influenza vaccination would facilitate efforts to contain COVID-19 outbreaks [2]. In addition, there is some evidence to suggest that patients with a recent history of influenza or influenza-like illnesses are at risk of more severe Covid-19 [3]. However, increasing, or even maintaining, levels of influenza vaccination may be problematic if reduced uptake patterns seen already in other vaccines also hold for the influenza vaccine. For example, the uptake of the measles-mumps-rubella (MMR) vaccine in England became 19.8% lower in the 3 weeks after full physical distancing measures were introduced in March than it was for the same period in 2019 [4].

The influenza season in the UK runs from December until March each year and the national vaccination program starts in September. At the time of data collection, eligibility for the free vaccine through the National Health Service (NHS) was the same as in previous years, being available to children aged two to eleven, adults over 65, pregnant women, health and social care workers, individuals aged 6 months to 65 years who are in clinical at-risk groups (many of which coincide with the COVID-19 at-risk groups), those living in a residential or nursing home, and anyone who is the main carer of an older or disabled person. In November 2020, the 2020-2021 influenza vaccination programme was extended to all adults aged 50 or older as well as to anyone living with someone who is at high risk from coronavirus. The vaccination is also available privately for a charge through primary care and pharmacies to the rest of the population. Despite the wide availability of a free vaccine for eligible individuals, uptake varies across the different categories of eligibility; for example, in the 2019–2020 season 72.4% of 65+ adults in England were vaccinated compared to 44.9% of individuals aged 6 months to 65 in clinical at-risk groups [5].

In order to protect people ahead of and during the annual influenza season, it might be helpful to understand intention to have a seasonal influenza vaccination during the COVID-19 pandemic. To

For peer review only - http://bmjopen.bmj.com/site/about/guidelines.xhtml

this end, we explored participants' likelihood of having the seasonal influenza vaccination as part of a larger cross-sectional study investigating attitudes towards a potential COVID-19 vaccination [6]. Previous research exploring factors associated with seasonal influenza vaccination uptake has identified a range of factors that might influence seasonal influenza uptake [7,8]. In this study we focused on socio-demographic factors such as age, ethnicity, and gender, general attitude towards vaccination, fear of needles, and past behavior (whether individuals had previously had a seasonal influenza vaccination). We explored these factors in participants who were eligible for the free influenza vaccination under pre-COVID criteria (in place at the time of data collection) and conducted a sensitivity analysis in which we included all those who were eligible under the pandemic-motivated broadened criteria.

Materials and Methods

Ethical approval for this study was granted by Keele University's Research Ethics Committee (reference: PS-200129). A nationally representative quota sample of 1500 UK adults (quotas set on age, gender and ethnicity) were recruited through Prolific's online research panel to complete a cross-sectional survey between 14th and 17th July 2020. Participants were included in this study if they were eligible to receive the free influenza vaccine through the NHS at the time of data collection (aged 65 years or over, pregnant, working in health or social care, or in a clinical risk group).[1] After providing consent, participants were asked to complete the survey, which included: sociodemographic questions (e.g., age, gender, ethnicity, employment status, highest educational or professional qualification); clinical questions (e.g., whether they or someone else in their household (if applicable) had a chronic illness that made them clinically vulnerable to serious illness from COVID-19); questions about COVID-19 (e.g., whether they were worried about catching coronavirus); and questions about a possible COVID-19 vaccination (e.g., whether they thought most people would get a coronavirus vaccination). We also asked participants to what extent they agreed that 'in general, vaccination is a good thing' and to what degree they were 'afraid of needles' (both on an eleven-point scale from "strongly disagree" to "strongly agree"), and if they had been vaccinated for seasonal influenza last winter (yes/no). The outcome measure for this study, influenza vaccination intention, was measured by asking participants how likely they would be to have the seasonal influenza vaccine "this winter" (eleven-point scale, from "extremely unlikely" to "extremely likely").

[1] We did not collect data on whether participants were living in a care home or whether they were a main carer and so these eligibility criteria are not explicitly represented in our analysis.

For peer review only - http://bmjopen.bmj.com/site/about/guidelines.xhtml

Full details of the wider study, including survey methodology, are reported elsewhere [6]. The survey is available in online supplemental file 1.

Since eligibility for the free influenza vaccine in the 2020-2021 season was widened after data collection, as a sensitivity analysis, we re-ran analyses using these broader criteria.

Statistical analysis

In order to identify factors associated with intention to receive the seasonal influenza vaccine in the 2020–2021 season, we used a multivariable logistic regression model, based on those respondents expressing a clear intention either to have nor not to have the vaccine. The predictors in the model were specified *a priori*, based on previous research [7,8]: sociodemographic variables; uptake of influenza vaccine last winter; and beliefs about vaccination (value of vaccination in general; afraid of needles); see Table 1. Odds ratios with 95% confidence intervals are reported, adjusted for all of the other predictors in the model; in addition, the corresponding crude (bivariate) odds ratios are given for the purpose of comparison. We used the Nagelkerke pseudo-R^2 statistic to express the goodness-of-fit of the model. Statistical significance was set at $p \leq .05$.

Patient and public involvement

Patients or the public were not involved in the design, or conduct, or reporting or dissemination plans of this research.

Results

At the time of data collection, 570 individuals in our sample were eligible for the free influenza vaccine. The distribution of influenza vaccination intention was bimodal, with the majority of responses clustering at both ends of the scale. We therefore dichotomized this variable as 0–2 = 'no' (*n* = 126; 22.1%) and 8–10 = 'yes' (*n* = 340; 59.7%) on the 0–10 scale. The 466 respondents who expressed a clear intention either to have or not to have the seasonal influenza vaccine were included in the analysis and the 104 (18.2%) indeterminate cases were not analysed further. The results of the regression analysis are shown in Table 2.

Table 1. Characteristics and attitudes of those respondents who expressed a clear intention either to have or not to have the influenza vaccine (*n*=466). Values are *n* (%) except where stated otherwise.

**Variable**	
Age (years): mean (SD); range	53.07 (16.86); 18–87
Gender[a]	

For peer review only - http://bmjopen.bmj.com/site/about/guidelines.xhtml

Male	203 (43.7)
Female	262 (56.3)
Ethnicity[b]	
White	403 (87.0)
Non-white	60 (13.0)
Qualifications	
Degree equivalent or higher	231 (49.6)
Other	235 (50.4)
Working[c]	
Part-time	58 (12.5)
Full-time	136 (29.3)
Not working/other[d]	270 (58.2)
Key worker	
Yes	175 (37.6)
No	291 (62.4)
Influenza vaccination last winter[a]	
Yes	299 (64.3)
No	166 (35.7)
In general, vaccination is a good thing (0–10 scale): mean (SD)[e]	8.78 (2.09)
I am afraid of needles (0–10 scale): mean (SD)[e]	2.14 (3.17)

[a] 1 missing value; [b] 3 missing values; [c] 2 missing values; [d] includes 30 unemployed, 36 furloughed and 157 retired; [e] these variables showed a marked skew.

For peer review only

Table 2. Logistic regression analysis of variables associated with intention to receive a seasonal influenza vaccine. Reference categories for the odds ratios are shown where appropriate. Confidence intervals and *p* values relate to the adjusted odds ratios.

	**Adjusted (crude) odds ratio**	**95% confidence interval**	***p* value**
Age (years)	1.02 (1.03)	0.99, 1.04	.190

Gender (reference: female)	1.24 (1.37)	0.60, 2.59	.565
Ethnicity – white (reference: black and minority ethnic)	0.60 (1.99)	0.23, 1.53	.281
Qualifications – degree equivalent or higher (reference: other)	1.52 (0.94)	0.72, 3.22	.278
Working (reference: not working/other)			
Part-time	1.12 (0.75)	0.39, 3.21	.830
Full-time	1.51 (0.81)	0.64, 3.56	.342
Key worker (reference: not key worker)	0.59 (0.62)	0.27, 1.31	.197
Influenza vaccination last winter (reference: no vaccination)	278.58 (273.58)	78.04, 994.46	<.001
In general, vaccination is a good thing (0–10 scale)	1.45 (1.57)	1.19, 1.77	<.001
I am afraid of needles (0–10 scale)	0.98 (.091)	0.89, 1.09	.757

n = 460 (6 cases with missing data on one or more variables were not analyzed). Nagelkerke R^2 = .760.

A positive attitude to vaccination and previous vaccination behavior were significant predictors of intention to have the influenza vaccine in the 2020–2021 season. As indicated by the large odds ratio, previous influenza vaccination behavior was a markedly stronger predictor.

Sensitivity analysis

We conducted the above analyses based on participants who were eligible for the free influenza vaccine at the time of data collection. In the intervening time, these criteria were broadened. Using the broadened eligibility criteria, 1003 respondents were eligible in 2020–2021. Of these, 491 (49.0%) respondents expressed a clear intention to have the vaccine, 291 (29.0%) a clear intention not to have the vaccine, and there were 221 (22.0%) indeterminate cases. As before, the indeterminate cases were not analyzed. The results of the sensitivity analysis are shown in Table 3.

Table 3. Logistic regression analysis of variables associated with intention to receive a seasonal influenza vaccine, using the broadened eligibility criteria introduced in the 2020–2021 season. Reference categories for the odds ratios are shown where appropriate. Confidence intervals and *p* values relate to the adjusted odds ratios.

	**Adjusted (crude) odds ratio**	**95% confidence interval**	***p* value**
Age (years)	1.02 (1.02)	1.00, 1.04	.046

For peer review only - http://bmjopen.bmj.com/site/about/guidelines.xhtml

Gender (reference: female)	1.24 (1.12)	0.76, 2.01	.388
Ethnicity – white (reference: black and minority ethnic)	0.75 (1.73)	0.36, 1.56	.443
Qualifications – degree equivalent or higher (reference: other)	1.00 (1.02)	0.62, 1.59	.983
Working (reference: not working/other)			
Part-time	0.80 (0.68)	0.40, 1.58	.519
Full-time	1.03 (0.71)	0.58, 1.81	.929
Key worker (reference: not key worker)	0.93 (0.83)	0.55, 1.57	.776
Influenza vaccination last winter (reference: no vaccination)	281.78 (262.85)	95.35, 832.72	<.001
In general, vaccination is a good thing (0–10 scale)	1.54 (1.57)	1.31, 1.81	<.001
I am afraid of needles (0–10 scale)	0.98 (0.92)	0.91, 1.05	.529

n = 774 (8 cases with missing data on one or more variables were not analyzed). Nagelkerke R^2 = .716.

Compared to the main analysis, a somewhat smaller percentage of respondents indicated a clear intention to be vaccinated (49.0% versus 59.7%), and correspondingly a larger percentage not to be vaccinated (29.0% versus 22.1%). However, the odds ratios were of a similar magnitude to those in the main analysis and with similar associated *p* values (though with the larger sample size, the odds ratio for age became significant), suggesting that whilst the broader eligibility criteria may influence the percentage of individuals intending to be vaccinated against influenza, they have little effect on the predictors of such vaccination behavior.

In both the main analysis and the sensitivity analysis, the adjusted and crude odds ratios were similar, with the exception of those for ethnicity. For this variable, the odds ratios changed noticeably (and went from being significant to non-significant) after adjustment for the other predictors in the multivariable model, suggesting that some of the explanatory effect of ethnicity was redistributed to other predictors in the full model.

Discussion

These findings strongly suggest that individuals who had the influenza vaccine in the last influenza season were likely to intend to have it again in the 2020-2021 season. This is consistent with findings from the H1N1 influenza pandemic [7] as well as with findings from studies exploring influenza vaccination intentions during the COVID pandemic in other countries and regions such as Italy [9,10] and Catalonia [11]. It also aligns with the finding that across six countries (US, Canada, Israel, Japan,

For peer review only - http://bmjopen.bmj.com/site/about/guidelines.xhtml

Spain, Switzerland) parents' intention to vaccinate their child against seasonal influenza was influenced by their and their child's previous influenza vaccination status [12]. However, there are still key issues to address. Vaccination intention across all those individuals in our sample who were eligible for the vaccine (59.7%) at the time of data collection was slightly lower than the reported uptake from the last influenza season (64.3%). Furthermore, it is likely that actual uptake will be lower than intention as a result of the intention-behavior gap [13], making it important that efforts are made to convert positive intentions into uptake. This might be achieved through appropriate messaging and special arrangements for vaccine delivery, particularly for those who might be shielding or at higher risk from COVID-19 and reluctant to attend their GP surgery. Both approaches are also likely to be needed to motivate those individuals who have not previously had the influenza vaccine and those individuals who are eligible for free vaccination but who were among respondents in our sample who indicated they definitely did not intend to be vaccinated (22.1%) or were unsure (18.2%).

Limitations of the current study include that participants were reporting intention to be vaccinated rather than actual vaccination status and that they were collected before a COVID-19 vaccination was a reality. However, since public health systems globally are likely to be managing seasonal influenza against a backdrop of COVID-19 for the foreseeable future, we believe these data provide useful information to assist with understanding the evolving response to national vaccination programs.

The NHS is often overwhelmed during the influenza season, needing, for example, to cancel routine operations. The extension of the influenza vaccination program in 2020-2021 to people aged 50 years and over and to those living with someone who is at high risk from coronavirus may also help decrease the burden of influenza season. Potential carry-over effects into the next influenza season (2021-2022 in the UK) in light of the current availability of COVID-19 vaccination; and ongoing public health and media discussions regarding the need for seasonal vaccination programs for both corona and influenza viruses suggest that the current dataset can be used as a baseline for future evaluation of the uptake of the influenza vaccination within an ever changing context. Increasing uptake of the seasonal influenza vaccine in a timely fashion will relieve pressure on health services. If this is to be successful, strategies to achieve this increase need to be designed now.

For peer review only

Data statement: Survey items and dataset are available from: DOI 10.17605/OSF.IO/94856.

Conflict of interest: NS is the director of the London Safety and Training Solutions Ltd, which offers training in patient safety, implementation solutions and human factors to healthcare organisations and the pharmaceutical industry. The other authors have no conflicts of interest to declare.

Funding statement: Data collection was funded by a Keele University Faculty of Natural Sciences Research Development award to SS, JS and NS, and a King's Together Rapid COVID-19 award granted jointly to LS, GJR, RA, NS, SS and JS. LS, RA and GJR are supported by the National Institute for Health Research Health Protection Research Unit (NIHR HPRU) in Emergency Preparedness and Response, a partnership between Public Health England, King's College London and the University of East Anglia. NS' research is supported by the National Institute for Health Research (NIHR) Applied Research Collaboration (ARC) South London at King's College Hospital NHS Foundation Trust. NS is a member of King's Improvement Science, which offers co-funding to the NIHR ARC South London and comprises a specialist team of improvement scientists and senior researchers based at King's College London. Its work is funded by King's Health Partners (Guy's and St Thomas' NHS Foundation Trust, King's College Hospital NHS Foundation Trust, King's College London and South London and Maudsley NHS Foundation Trust), Guy's and St Thomas' Charity and the Maudsley Charity. The views expressed are those of the authors and not necessarily those of the NIHR, the charities, Public Health England or the Department of Health and Social Care.

Author Contributions

Conceptualization, LS; Methodology, RA, MC, HD, GJR, NS, SS, JS, LS; Software, SS; Formal Analysis, JS; Writing – Original Draft Preparation, SS; Writing – Review & Editing, RA, MC, HD, GJR, NS, SS, JS, LS; Funding Acquisition, RA, GJR, NS, SS, JS, LS.

For peer review only

References

1. Maltezou HC, Theodoridou K, Poland G. Influenza immunization and COVID-19. *Vaccine* 2020;38(39):6078–9.

2. Li Q, Tang B, Bragazzi NL, Xiao Y, Wu J. Modeling the impact of mass influenza vaccination and public health interventions on COVID-19 epidemics with limited detection capability. *Math Biosci* 2020;325:108378.

3. Ceccarelli G, d'Ettorre G, Innocenti GP, Mastroianni CM, Ciccozzi M, d'Ettorre G. Is previous influenza-like illness a potential Trojan horse for COVID-19? *Crit Care* 2020;24(1):1–2.

4. McDonald HI, Tessier E, White JM, Woodruff M, Knowles C, Bates C, et al. Early impact of the coronavirus disease (COVID-19) pandemic and physical distancing measures on routine childhood vaccinations in England, January to April 2020. *Eurosurveillance* 2020;25(19):2000848.

5. Public Health England (2020). *Surveillance of influenza and other respiratory viruses in the UK: Winter 2019 to 2020.* London: Public Health England.

6. Sherman SM, Smith LE, Sim J, Amlôt R, Cutts M, Dasch H, Rubin GR, Sevdalis N. COVID-19 vaccination intention in the UK: results from the COVID-19 Vaccination Acceptability Study (CoVAccS), a nationally representative cross-sectional survey. *Hum Vaccin Immunother* 2020; DOI: 10.1080/21645515.2020.1846397

7. Bish A, Yardley L, Nicoll A, Michie S. Factors associated with uptake of vaccination against pandemic influenza: a systematic review. *Vaccine* 2011;29(38):6472–84.

8. Wheelock A, Miraldo M, Thomson A, Vincent C, Sevdalis N. Evaluating the importance of policy amenable factors in explaining influenza vaccination: a cross-sectional multinational study. *BMJ Open* 2017;7:e014668

9. Pastorino R, Villani L, Mariani M, Ricciardi W, Graffigna G, Boccia S. Impact of COVID-19 Pandemic on Flu and COVID-19 Vaccination Intentions among University Students. *Vaccines* 2021;9(2):70.

10. Domnich A, Cambiaggi M, Vasco A, Maraniello L, Ansaldi F, Baldo V, Bonanni P, Calabrò GE, Costantino C, de Waure C, Gabutti G. Attitudes and beliefs on influenza vaccination during the COVID-19 pandemic: results from a representative Italian survey. *Vaccines* 2020;8(4):711.

For peer review only

11. Bonet-Esteve A, Muñoz-Miralles R, Gonzalez-Claramunt C, Rufas AM, Cruz XP, Vidal-Alaball J. Influenza vaccination during the coronavirus epidemic: intention to Vaccinate among the at-risk Population in the Central Catalonia Health Region (VAGCOVID). *Research Square* 2021;pre-print.

12. Goldman RD, McGregor S, Marneni SR, Katsuta T, Griffiths MA, Hall JE, Seiler M, Klein EJ, Cotanda CP, Gelernter R, Hoeffe J. Willingness to vaccinate children against influenza after the Coronavirus disease 2019 pandemic. *J Pediatr* 2021;228:87-93.

13. Sniehotta FF, Scholz U, Schwarzer R. Bridging the intention–behaviour gap: planning, self-efficacy, and action control in the adoption and maintenance of physical exercise. *Psychol Health* 2005;20(2):143–60.

For peer review only

For peer review only - http://bmjopen.bmj.com/site/about/guidelines.xhtml

CoVAccS Survey 1 - Final

Start of Block: Information about the research

Q1.2 **Covid-19 Vaccination Acceptability Study – CoVAccS**
Survey exploring public attitudes towards a possible coronavirus (Covid-19) vaccination **Participant Information** We are interested in understanding how people feel about a possible coronavirus (Covid-19) vaccination and whether you would be likely to have the vaccine if one becomes available to you. We would be very grateful if you would complete this 3-part survey. The first part contains some questions about you, the second part contains questions about the coronavirus illness, the third part contains questions about a possible coronavirus vaccination, whether you would have such a vaccination and some general questions about your thoughts regarding vaccination and healthcare. To assist us with this, we would be grateful if you would complete this survey. It should take no more than 20 minutes. Please note that all responses will be anonymous. What will I need to do? If you decide to take part, you should tick the consent box at the bottom of this page and then complete the questionnaire. Please ensure that you have answered all questions on a page before moving on to the next one. Please note that once you have completed the final page you will be unable to withdraw your data as it is anonymous. Will my taking part in the study be kept confidential? Yes. The survey is anonymous (we don't ask for your name or any identifying information). What will happen to the results of the research study? We intend to publish the results of this study in scientific journals. We will also present it at scientific conferences and we may also pass the results of the research to relevant policy makers. This will contribute to the debate around vaccination policy. You will not be identified in any research presentation or publication as the study is completed anonymously. The data you have provided will be stored on a password protected computer and laptop. On completion of the project the data will be stored indefinitely in an online repository such as the Open Science Framework to which access will be open. You will not be identifiable from the data since your participation is entirely anonymous. Who is conducting the research? The research is being conducted by a team from Keele University, King's College London, and Public Health England. Who has reviewed the study? All research conducted by Keele University is looked at by an independent group of people, called a Research Ethics Committee, to protect your interests. This study has been reviewed and approved by Keele's Research Ethics Committee. What if there is a problem? If you have a concern about any aspect of this study, you may wish to speak to the principal investigator, Dr Sue Sherman, at Keele University at s.m.sherman@keele.ac.uk. Alternatively, you may wish to contact Dr Joseph Brooks (j.l.brooks@keele.ac.uk) who is the Director of Research in the School of Psychology at Keele University.

For peer review only

Q1.3
Consent agreement If you are happy to take part in this survey, please read the following statements: I confirm that I have read and understand the information above for this study I understand that my data will be anonymous and that my participation is voluntary I understand that I have the right to withdraw from the study at any time by closing the survey I agree to allow the data collected to be used for research projects related to this project Now please click on one of the options below:

○ I agree with the statements above and I am happy to take part in this study (1)

○ I do not wish to take part in this study (2)

End of Block: Information about the research

Start of Block: Does not consent

Q2.1
As you do not wish to participate in this study, please return your submission on Prolific by selecting the 'Stop without completing' button.

End of Block: Does not consent

Start of Block: Prolific ID

Q3.2
Before you start, please:

maximize your browser window;
switch off phone/e-mail/music and anything else distracting
and please enter your Prolific ID in the box below [it can be found at the top of this webpage or when going to your account info]:

____

End of Block: Prolific ID

Start of Block: Age check

For peer review only - http://bmjopen.bmj.com/site/about/guidelines.xhtml
For peer review only

Q4.2 How old are you?

- ◯ 17 or younger (1)
- ◯ 18 (2)
- ◯ Then options through to
- ◯ 100 (84)
- ◯ Older than 100 (104)

End of Block: Age check

Start of Block: Not eligible

Q5.1 Unfortunately you are not eligible to take part in this survey as you are under 18, please return your submission on Prolific by selecting the 'Stop without completing' button.

End of Block: Not eligible

Start of Block: Demographics

Q6.2 **PART 1: INFORMATION ABOUT YOU**

Q6.3 How would you describe your gender?

- ◯ Female (0)
- ◯ Male (1)
- ◯ Non-binary (2)
- ◯ Prefer to self-describe (please specify) (3) ____
- ◯ Prefer not to say (-7)

For peer review only - http://bmjopen.bmj.com/site/about/guidelines.xhtml

Q6.4 Please choose one of the options below which best describes your ethnic group or background. These categories reflect the categories used in the Census 2011.

◯ English/ Welsh/ Scottish/ Northern Irish/ British (1)

◯ Irish (2)

◯ Gypsy or Irish traveller (3)

◯ Any other white background, please specify (4)

____

◯ White & Black Caribbean (5)

◯ White and Black African (6)

◯ White and Asian (7)

◯ Any Other Mixed background, please specify (8)

____

◯ Indian (9)

◯ Pakistani (10)

◯ Bangladeshi (11)

◯ Chinese (12)

◯ Any other Asian background, please specify (13)

____

◯ African (14)

◯ Caribbean (15)

◯ Any other Black background, please specify (16)

____

◯ Arab (17)

For peer review only - http://bmjopen.bmj.com/site/about/guidelines.xhtml

◯ Any other ethnic group, please specify (18)

____

◯ Prefer not to say (-7)

Page Break

Q6.6 What is your religion?

◯ No religion (1)

◯ Christian (2)

◯ Buddhist (3)

◯ Hindu (4)

◯ Jewish (5)

◯ Muslim (6)

◯ Sikh (7)

◯ Any other religion, please describe (8)

____

◯ Prefer not to say (-7)

For peer review only

For peer review only - http://bmjopen.bmj.com/site/about/guidelines.xhtml

Q6.7 What is the highest level of educational or professional qualification you have received?

- No formal qualifications (1)
- Youth training certificate/skillseekers (2)
- Recognised trade apprenticeship (3)
- Clerical and commercial (4)
- City & Guilds certificate (5)
- City & Guilds certificate – advanced (6)
- ONC (7)
- CSE grades 2–5 (8)
- CSE grade 1, GCE O level, GCSE, School Certificate (9)
- Scottish Ordinary/ Lower Certificate (10)
- GCE A level or Higher Certificate (11)
- Scottish Higher Certificate (12)
- Nursing or midwifery qualification (e.g. SEN, SRN, SCM, RGN) (13)
- Teaching qualification (not degree) (14)
- University diploma (15)
- University or CNAA first degree (e.g. BA, BSc, BEd) (16)
- University or CNAA higher degree (e.g. MSc, PhD) (17)
- Other technical, professional or higher qualification (18)
- Don't know (19)

For peer review only

◯ Prefer not to say (-7)

Q6.8 Which ONE of the following best describes your current working situation?

◯ Working full time (30 hours per week or more) (1)

◯ Usually working full time (30 hours per week or more), but currently furloughed (2)

◯ Working part time (8–29 hours per week) (3)

◯ Usually working part time (8–29 hours per week), but currently furloughed (4)

◯ Stay-at-home parent (5)

◯ Unemployed (6)

◯ Retired (7)

◯ Student (8)

◯ Other (9)

◯ Don’t know (10)

◯ Prefer not to say (-7)

For peer review only

Q6.9 In which of the following categories would you place your total household income from all sources before tax and any other deductions?

- ◯ Under £10,000 (1)
- ◯ £10,000–£19,999 (2)
- ◯ £20,000–£29,999 (3)
- ◯ £30,000–£39,999 (4)
- ◯ £40,000–£49,999 (5)
- ◯ £50,000–£74,999 (6)
- ◯ £75,000 or over (7)
- ◯ Don't know (8)
- ◯ Prefer not to say (-7)

Page Break

For peer review only

Q6.11 Where in the UK do you live?

- ◯ East Midlands (1)
- ◯ East of England (2)
- ◯ London (3)
- ◯ North East (4)
- ◯ North West (5)
- ◯ Northern Ireland (6)
- ◯ Scotland (7)
- ◯ South East (8)
- ◯ South West (9)
- ◯ Wales (10)
- ◯ West Midlands (11)
- ◯ Yorkshire and the Humber (12)
- ◯ Prefer not to say (-7)

For peer review only

Q6.12 How many people live in your household including yourself?

- ◯ 1 (1)
- ◯ 2 (2)
- ◯ 3–4 (3)
- ◯ 5–6 (4)
- ◯ 7 or more (5)
- ◯ Prefer not to say (-7)

Q6.13 The colour test is simple, when asked for your favourite colour you must enter the word purple in the text box below.

Based on the text you read above, what colour have you been asked to enter?

____

Q6.14 Do any of the following apply to you or someone else in your household?

	You			Someone else in your household		
	Yes (1)	No (0)	Prefer not to say (-7)	Yes (1)	No (0)	Prefer not to say (-7)

For peer review only

For peer review only - http://bmjopen.bmj.com/site/about/guidelines.xhtml

Received a letter from the NHS recommending that extra precautions against coronavirus are taken (this is called 'shielding') (Q6.11_1)	○	○	○	○	○	○
A lung condition (such as asthma, COPD, emphysema or bronchitis) (Q6.11_2)	○	○	○	○	○	○
Heart disease (such as heart failure) (Q6.11_3)	○	○	○	○	○	○
Chronic kidney disease (Q6.11_4)	○	○	○	○	○	○
Liver disease (such as hepatitis) (Q6.11_5)	○	○	○	○	○	○
A condition affecting the brain or nerves (such as Parkinson's disease, motor neurone disease, multiple sclerosis or cerebral palsy) (Q6.11_6)	○	○	○	○	○	○
Diabetes (Q6.11_7)	○	○	○	○	○	○

For peer review only

For peer review only - http://bmjopen.bmj.com/site/about/guidelines.xhtml

A condition that means there is a high risk of getting infections (Q6.11_8)	○	○	○	○	○	○
Taking medicine that can affect the immune system (such as steroids) (Q6.11_9)	○	○	○	○	○	○
Classified as very obese (a body mass index (BMI) of 40 or above) (Q6.11_10)	○	○	○	○	○	○
Pregnant (Q6.11_11)	○	○	○	○	○	○

For peer review only

Q6.15 Do any of the following apply to you?

	You		
	Yes (1)	No (0)	Prefer not to say (-7)

For peer review only - http://bmjopen.bmj.com/site/about/guidelines.xhtml

Received a letter from the NHS recommending that extra precautions against coronavirus are taken (this is called 'shielding') (Q6.12_1)	○	○	○
A lung condition (such as asthma, COPD, emphysema or bronchitis) (Q6.12_2)	○	○	○
Heart disease (such as heart failure) (Q6.12_3)	○	○	○
Chronic kidney disease (Q6.12_4)	○	○	○
Liver disease (such as hepatitis) (Q6.12_5)	○	○	○
A condition affecting the brain or nerves (such as Parkinson's disease, motor neurone disease, multiple sclerosis or cerebral palsy) (Q6.12_6)	○	○	○
Diabetes (Q6.12_7)	○	○	○
A condition that means there is a high risk of getting infections (Q6.12_8)	○	○	○
Taking medicine that can affect the immune system (such as steroids) (Q6.12_9)	○	○	○
Classified as very obese (a body mass index (BMI) of 40 or above) (Q6.12_10)	○	○	○

For peer review only

For peer review only - http://bmjopen.bmj.com/site/about/guidelines.xhtml

Pregnant (Q6.12_11)	○	○	○

Page Break

Q6.17 Please could you indicate if you work in any of the following sectors or roles? Please include any voluntary work. Please tick all that apply.

▢ Health or social care (e.g. doctors, nurses, midwives, paramedics, social workers, care workers; or work as part of the health and social care supply chain, including producers and distributers of medicines and medical equipment) (1)

▢ Education and childcare (e.g. teaching and support staff, childminders, social workers, specialist education professionals) (2)

▢ Key public services (e.g. the justice system, religious staff, charities delivering frontline services, journalists, broadcasters, undertakers) (3)

▢ Local and national government in a role essential to continuous provision of essential services (e.g the payment of benefits, or processing of new benefit applications) (4)

▢ Food and essential goods (e.g. food production, processing, distribution, sale and delivery, as well as those essential to the provision of other key goods such as hygienic or veterinary medicine) (5)

▢ Public safety and national security (e.g. police and support staff, Ministry of Defence civilians, contractor and armed forces, fire and rescue service employees, National Crime Agency staff, border security staff, prison and probation staff and other national security roles) (6)

▢ Transport (e.g. air, water, road and rail passenger and freight transport modes) (7)

▢ Utilities, communication and financial services (e.g. banks, building societies and financial market infrastructure; the oil, gas, electricity and water sectors; information technology and data infrastructure sector; civil nuclear, chemicals, telecommunications, network operations, field engineering, call centre staff, IT and data infrastructure, 999 and

For peer review only - http://bmjopen.bmj.com/site/about/guidelines.xhtml

111 critical services, postal services and delivery, payments providers and waste disposal) (8)

☐ None of the above (9)

☐ Prefer not to say (-7)

Q6.18 Last winter, did you have a vaccination for seasonal flu?

○ Yes (1)

○ No (0)

○ Don't know (2)

○ Prefer not to say (-7)

End of Block: Demographics

Start of Block: Pre-survey statement

Q7.2 Over the next few pages we will ask you a series of questions about the coronavirus illness and a possible vaccination. We are interested to know your personal opinion about these topics.

Please click on 'NEXT' when you are ready to continue.

End of Block: Pre-survey statement

Start of Block: Questions about Covid-19 the illness 1

Q8.2 **PART 2: YOUR THOUGHTS ABOUT THE CORONAVIRUS (COVID-19) ILLNESS**

Q8.3
To what extent do you think coronavirus poses a risk to people in the UK?

For peer review only

◯ Major risk (5)

◯ Significant risk (4)

◯ Moderate risk (3)

◯ Minor risk (2)

◯ No risk at all (1)

◯ Don't know (0)

Q8.4
To what extent do you think coronavirus poses a risk to you personally?

◯ Major risk (5)

◯ Significant risk (4)

◯ Moderate risk (3)

◯ Minor risk (2)

◯ No risk at all (1)

◯ Don't know (0)

For peer review only

For peer review only - http://bmjopen.bmj.com/site/about/guidelines.xhtml

Q8.5 Do you believe you have had, or currently have, coronavirus? (Please select the one option that BEST applies to you)

◯ I have definitely had it or definitely have it now (4)

◯ I have probably had it or probably have it now (3)

◯ I have probably not had it and probably don't have it now (2)

◯ I have definitely not had it and definitely don't have it now (1)

◯ Don't know (0)

◯ Prefer not to say (-7)

Q8.6 Do you personally know anyone (excluding yourself) who has had coronavirus?

◯ Yes (1)

◯ No (0)

◯ Don't know (0)

◯ Prefer not to say (-7)

End of Block: Questions about Covid-19 the illness 1

Start of Block: Questions about Covid-19 the illness 2

Q9.2 PART 2: YOUR THOUGHTS ABOUT THE CORONAVIRUS (COVID-19) ILLNESS

Q9.3 Please indicate the extent to which you agree or disagree with the following statements by ticking a number between 0 and 10, where **0 means 'strongly disagree' and 10 means 'strongly agree':**

For peer review only - http://bmjopen.bmj.com/site/about/guidelines.xhtml

Q9.4 I am worried about catching coronavirus
Q9.5 I believe that coronavirus would be a mild illness for me
Q9.6 Too much fuss is being made about the risk of coronavirus
Q9.7 We are all responsible for reducing the spread of the coronavirus
Q9.8 It's important that you pay attention to this study, so please select 0 ('strongly disagree') for this item
Q9.9 I believe I am immune to coronavirus
Q9.10 The coronavirus pandemic has had a big impact on my life
Q9.11 I trust the NHS to manage the coronavirus pandemic in the UK
Q9.12 I trust the Government to manage the coronavirus pandemic in the UK

End of Block: Questions about Covid-19 the illness 2

Start of Block: Questions about the Covid-19 vaccination 1

Q10.2 **PART 3: YOUR THOUGHTS ABOUT A CORONAVIRUS (COVID-19) VACCINATION**

For the following questions, please imagine that a coronavirus vaccine is widely available.

Q10.3 When a coronavirus vaccination becomes available to you, how likely is it that you will have one? Please select a number between 0 and 10, where **0 means 'extremely unlikely' and 10 means 'extremely likely'**:

End of Block: Questions about the Covid-19 vaccination 1

Start of Block: Questions about the Covid-19 vaccination 2

Q11.2 **PART 3: YOUR THOUGHTS ABOUT A CORONAVIRUS (COVID-19) VACCINATION**

For the following questions, please imagine that a coronavirus vaccine is widely available.

Q11.3 Please indicate the extent to which you agree or disagree with the following statements by ticking a number between 0 and 10, where **0 means 'strongly disagree' and 10 means 'strongly agree':**

For peer review only

Q11.4 A coronavirus vaccination should be mandatory for everyone who is able to have it
Q11.5 Without a coronavirus vaccine, I am likely to catch coronavirus
Q11.6 If I get a coronavirus vaccination, I will be protected against coronavirus
Q11.7 If I don't get a coronavirus vaccination and end up getting coronavirus, I would regret not getting the vaccination
Q11.8 It would be very easy for me to have a coronavirus vaccination
Q11.9 A coronavirus vaccination could give me coronavirus
Q11.10 It's important that you pay attention to this study, so please select 10 ('strongly agree') for this item
Q11.11 I would be worried about experiencing side effects from a coronavirus vaccination

End of Block: Questions about the Covid-19 vaccination 2

Start of Block: Questions about the Covid-19 vaccination 3

Q12.2 PART 3: YOUR THOUGHTS ABOUT A CORONAVIRUS (COVID-19) VACCINATION

For the following questions, please imagine that a coronavirus vaccine is widely available.

Q12.3 Please indicate the extent to which you agree or disagree with the following statements by ticking a number between 0 and 10, where **0 means 'strongly disagree' and 10 means 'strongly agree':**

Q12.4 I might regret getting a coronavirus vaccination if I later experienced side effects from the vaccination
Q12.5 A coronavirus vaccination will be too new for me to be confident about getting vaccinated
Q12.6 Most people will get a coronavirus vaccination
Q12.7 Other people like me will get a coronavirus vaccination
Q12.8 In general, vaccination is a good thing
Q12.9 I am afraid of needles
Q12.10 If I were vaccinated, I think I would not need to follow social distancing and other restrictions for coronavirus
Q12.11 I know enough about the coronavirus illness to make an informed decision about whether or not to get vaccinated
Q12.12 I know enough about the coronavirus vaccine to make an informed decision about whether or not to get vaccinated

End of Block: Questions about the Covid-19 vaccination 3

Start of Block: Questions about the Covid-19 vaccination 4

For peer review only - http://bmjopen.bmj.com/site/about/guidelines.xhtml

Q13.2 PART 3: YOUR THOUGHTS ABOUT A CORONAVIRUS (COVID-19) VACCINATION

For the following questions, please imagine that a coronavirus vaccine is widely available.

Q13.3 Please indicate the extent to which you agree or disagree with the following statements by ticking a number between 0 and 10, where **0 means 'strongly disagree' and 10 means 'strongly agree':**

Q13.4 Only people who are at risk of serious illness from coronavirus need to be vaccinated
Q13.5 My family would approve of my having a coronavirus vaccination
Q13.6 My friends would approve of my having a coronavirus vaccination
Q13.7 If a coronavirus vaccination were recommended by the Government, I would get vaccinated
Q13.8 If a coronavirus vaccination were recommended by a health care professional (e.g. GP or nurse), I would get vaccinated
Q13.9 Widespread coronavirus vaccination is just a way to make money for vaccine manufacturers
Q13.10 A coronavirus vaccine will allow us to get back to 'normal'
Q13.11 There would be no point in having the coronavirus vaccination unless I could go back to my normal life

End of Block: Questions about the Covid-19 vaccination 4

Start of Block: Questions about the Covid-19 vaccination 5

Q14.2 PART 3: YOUR THOUGHTS ABOUT A CORONAVIRUS (COVID-19) VACCINATION

For the following questions, please imagine that a coronavirus vaccine is widely available.

Q14.3 As far as you know, would your employer want you to have the coronavirus vaccination?

- ◯ Yes (0)
- ◯ No (1)
- ◯ Don't know (2)
- ◯ Not applicable (11)

For peer review only

Q14.4 As far as you know, is there currently a widely-available vaccination to protect against coronavirus?

- ◯ Yes (1)
- ◯ No (0)
- ◯ Don't know (2)
- ◯ Prefer not to say (-7)

Q14.5
This winter, how likely is it that you will have the seasonal flu vaccination? Please select a number between 0 and 10, where **0 means 'extremely unlikely' and 10 means 'extremely likely'**:

End of Block: Questions about the Covid-19 vaccination 5

Start of Block: Thank you

Q15.2 Thank you for taking part in this survey.

If you have any questions about the survey, please contact Dr Sue Sherman (s.m.sherman@keele.ac.uk).

If you have questions about coronavirus (Covid-19), please visit the NHS website: https://www.nhs.uk/conditions/coronavirus-covid-19/ or the government website: https://www.gov.uk/coronavirus.

End of Block: Thank you

For peer review only
